# Gut–kidney axis in IgA nephropathy: mechanisms linking microbiota dysbiosis to immune dysregulation, Gd-IgA1 generation, and renal injury

**DOI:** 10.3389/fimmu.2026.1798014

**Published:** 2026-08-10

**Authors:** Zhen Mu, Chunxue You, Ting Wang, Yubin Xu, Zhencang Zheng, Zhenxiong Zhao

**Affiliations:** 1 Tianjin Key Laboratory of Agricultural Animal Breeding and Healthy Husbandry, College of Animal Science and Veterinary Medicine, Tianjin Agricultural University, Tianjin, China; 2 Taizhou Central Hospital (Taizhou University Hospital), Taizhou, Zhejiang, China; 3 School of Medicine, Taizhou University, Taizhou, Zhejiang, China

**Keywords:** *Akkermansia muciniphila*, Gd IgA1, gut microbiota, gut microbiota dysbiosis, gut-kidney axis, IgAN

## Abstract

IgA nephropathy (IgAN) is the most common primary glomerulonephritis worldwide, with a particularly high prevalence in Asia, and is a leading cause of end-stage renal disease (ESRD). The disease typically presents initially with hematuria and proteinuria, accompanied by progressive decline in renal function. The hallmark pathological feature of IgAN is mesangial IgA1 deposition, and aberrant production of galactose-deficient IgA1 (Gd-IgA1) is considered the key pathogenic driver. Studies have shown that Gd-IgA1 forms immune complexes (ICs) with autoantibodies, which deposit in the glomeruli, elicit inflammatory cascades, and lead to renal tissue injury. Emerging evidence indicates that gut microbiota dysbiosis is closely linked to the pathophysiology of IgAN. Such dysbiosis may promote disease progression by modulating immune responses and disrupting intestinal barrier integrity. Under healthy conditions, the gut microbiota supports host homeostasis through immune regulation and barrier maintenance, whereas dysbiosis may trigger systemic immune activation and contribute to the development of IgAN. This review delineates the relationship between gut microbiota and IgAN, explores the mechanisms of the gut–kidney axis and its relevance to IgAN pathogenesis, and highlights the therapeutic potential of intestinal interventions, including probiotics and fecal microbiota transplantation, for the treatment of IgAN. By synthesizing current evidence, this review aims to provide a theoretical foundation for future research and clinical translation in this field.

## 1 Introduction

IgA nephropathy (IgAN) is the most common primary glomerulonephritis in Asia and a significant contributor to end-stage renal disease (ESRD) worldwide (1–3). Clinically, hematuria and proteinuria often present as the initial signs, occurring either alone or in combination (4–6). These manifestations are commonly linked to a progressive decline in renal function (7). The hallmark of renal pathology is mesangial IgA1 deposition, which is frequently accompanied by complement activation and mesangial cell proliferation (5, 8, 9).

**Figure 1:**
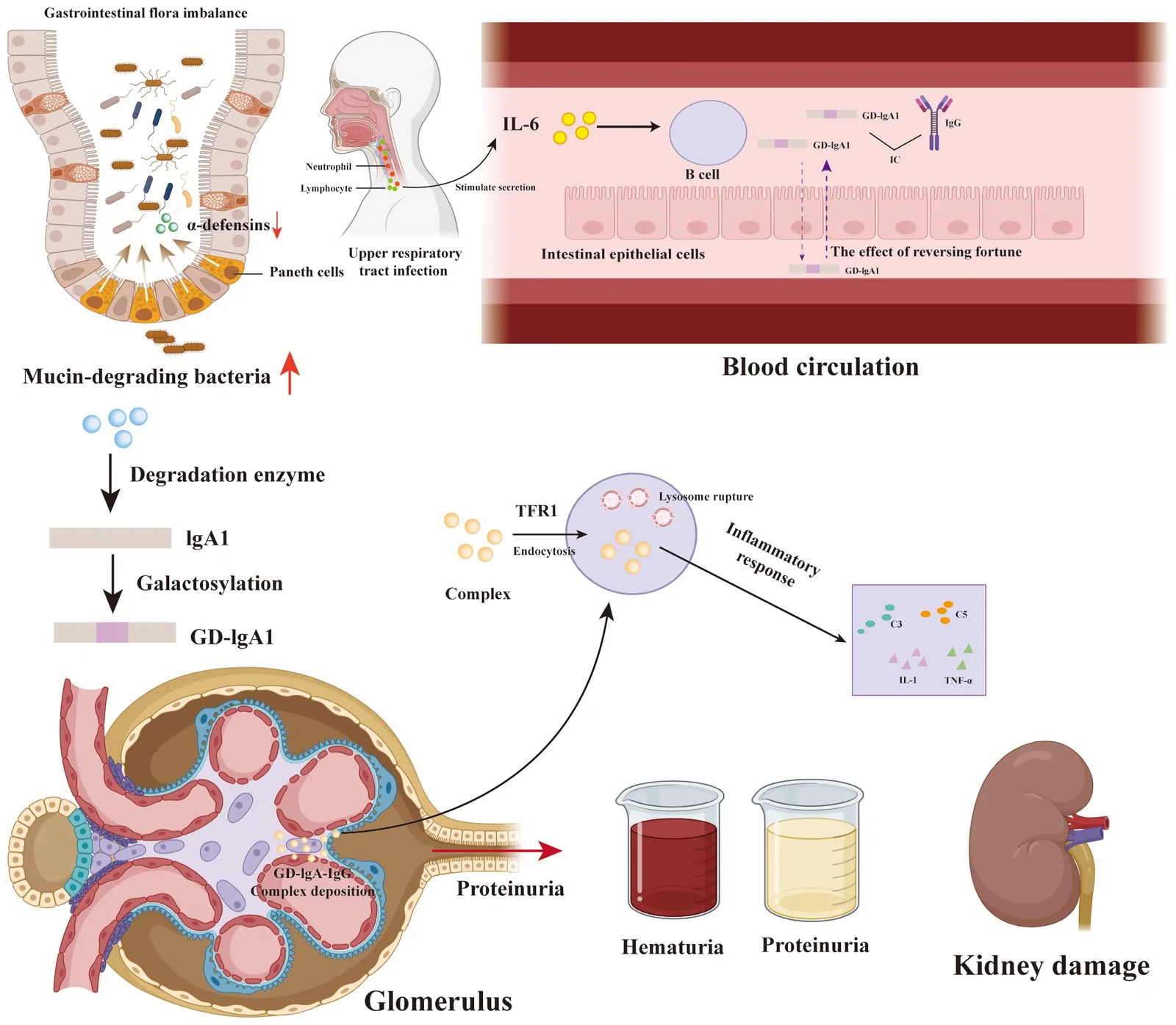
Gut–renal axis dysregulation in IgAN: from microbiota imbalance to renal injury. This schematic outlines a proposed pathogenic cascade linking gut microbiota dysbiosis and impaired mucosal defense to the generation of Gd-IgA1, immune-complex formation, and subsequent mesangial injury in IgAN. The dysbiosis observed in IgAN is characterized by an altered microbial composition and an expansion of mucus-degrading bacteria. Emerging evidence suggests that glycan-degrading enzymes from these bacteria may act on IgA1 molecules, promoting O-glycan deglycosylation and Gd-IgA1 production. Genome-wide association studies (GWAS) in IgAN have identified risk loci including the *DEFA* locus, suggesting a genetic contribution through impaired mucosal antimicrobial defense that may predispose individuals to dysbiosis and barrier dysfunction. Gd-IgA1 secreted from the intestinal mucosa enters the systemic circulation via retrotranscytosis, where it is recognized by circulating immunoglobulin G (IgG) autoantibodies, leading to the formation of pathogenic immune complexes. These immune complexes (ICs) accumulate in the glomerular mesangium and interact with transferrin receptor 1 (TfR1), triggering inflammatory cascades including complement activation and cytokine release, ultimately manifesting as hematuria, proteinuria, and progressive decline in renal function.

Galactose-deficient IgA1 (Gd-IgA1) is an aberrantly galactosylated isoform of IgA1, resulting from defective O-glycosylation in its hinge region (10). Numerous studies have consistently shown that serum Gd-IgA1 levels are significantly elevated in IgAN patients relative to healthy controls and patients with other renal diseases (11, 12). As a key pathogenic driver, Gd-IgA1 contributes to disease progression through two principal mechanisms. First, Gd-IgA1 acts as a self-antigen; its aberrant glyco-epitopes are recognized by circulating autoantibodies, leading to the formation of immune complexes (ICs) that deposit in the glomerular mesangium (12). Second, deposited Gd-IgA1-containing ICs trigger Toll-like receptor 4 (TLR4) signaling in mesangial cells refer to **Figure 1**, which induces lysosomal dysfunction and promotes the release of pro-inflammatory mediators, ultimately resulting in renal tissue injury (13, 14). A strong correlation has been established between circulating Gd-IgA1 levels and disease activity (P < 0.001), suggesting that Gd-IgA1 may serve as a useful biomarker for predicting the risk of progression to renal failure (13, 15).

The gut microbiota refers to the microbial communities residing in the intestinal tract, the composition of which is shaped by genetic, dietary, and environmental factors (16, 17). In healthy conditions, the gut microbiota maintains host homeostasis through multiple pathways. Microbial metabolites—such as short-chain fatty acids (SCFAs)—modulate mucosal immune responses by promoting IgA secretion and T-cell differentiation (18, 19); in parallel, commensal bacteria preserve intestinal barrier integrity by preventing pathogen colonization and reinforcing tight junctions between epithelial cells (20, 21). Conversely, when microbial diversity declines or certain pathogenic taxa expand, the mucosal barrier may become disrupted, leading to aberrant systemic immune activation and contributing to the development of autoimmune disorders and chronic inflammation (10).

The gut–kidney axis hypothesis underscores the substantial pathological cross talk between the gastrointestinal tract and the kidneys. In IgAN, gut microbiota dysbiosis drives disease progression through multiple interconnected pathways. One such pathway involves compositional shifts in the microbiota, such as expansion of mucin-degrading bacteria, which stimulate mucosal B lymphocytes and promote excessive Gd-IgA1 production (2, 3, 22). In parallel, enzymes from certain bacterial strains can directly cleave the glycan moieties of IgA1, thereby unmasking cryptic antigenic epitopes that are subsequently recognized by autoantibodies, leading to the formation of pathogenic ICs (10). Moreover, dysbiosis impairs intestinal barrier integrity and increases its permeability, which promotes retrotranscytosis of modified IgA1 from intestinal epithelial cells into the bloodstream, ultimately culminating in glomerular deposition (10, 20, 23). Supporting these mechanistic links, experimental studies have shown that transferring gut microbiota from IgAN patients into recipient mice elicits IgA deposition in the murine glomeruli (22, 24).

At present, several key scientific challenges remain unresolved in the field of gut microbiota and IgAN research. First, the issue of causality remains contentious. Although cohort studies have shown reduced α diversity and marked enrichment of certain bacterial genera in IgAN patients, it is still unclear whether gut dysbiosis acts as a driving factor of the disease or merely reflects its progression (3, 25, 26). Second, mechanistic investigations are still insufficient, and it is not well understood how the gut microbiota specifically influences Gd-IgA1 glycosylation—whether through TLR4 signaling or via its metabolic products (10, 22). Third, the therapeutic potential of microbiota-targeted interventions warrants further exploration. While probiotics and fecal microbiota transplantation (FMT) are proposed to slow IgAN progression by restoring the microbiota–immune axis, relevant clinical evidence remains limited (27–29). These unresolved issues substantially hinder the development of specific therapeutic strategies targeting the gut–kidney axis.

This review systematically synthesizes interdisciplinary evidence on the link between gut microbiota and IgAN, with three main objectives: first, to critically elucidate the mechanistic role of dysbiosis in driving Gd-IgA1-mediated pathogenesis; second, to evaluate the clinical relevance of microbiota-derived biomarkers, with special emphasis on the abundance of mucin-degrading bacteria; and third, to explore the translational promise of novel microbiota-targeted interventions, including FMT and prebiotics. Collectively, this paper aims to provide a theoretical foundation for future investigations in this rapidly evolving field.

## 2 Overview of the gut–kidney axis

The gut–kidney axis represents a bidirectional communication network that connects intestinal microbes, epithelial barrier function, host immunity, and renal homeostasis. Under healthy conditions, gut microbial metabolism supports barrier integrity and immune equilibrium, whereas in chronic kidney disease (CKD), impaired renal clearance and uremic toxicity are linked to intestinal barrier dysfunction and microbial dysbiosis, leading to increased systemic exposure to gut-derived metabolites and pro-inflammatory signals (30, 31). Recent work has underscored that these microbe–kidney interactions are functionally relevant across various renal disorders and has also identified important translational knowledge gaps that need to be addressed for microbiota-based therapeutic strategies (32–34).

### 2.1 Definition of the gut–kidney axis

**Figure 2:**
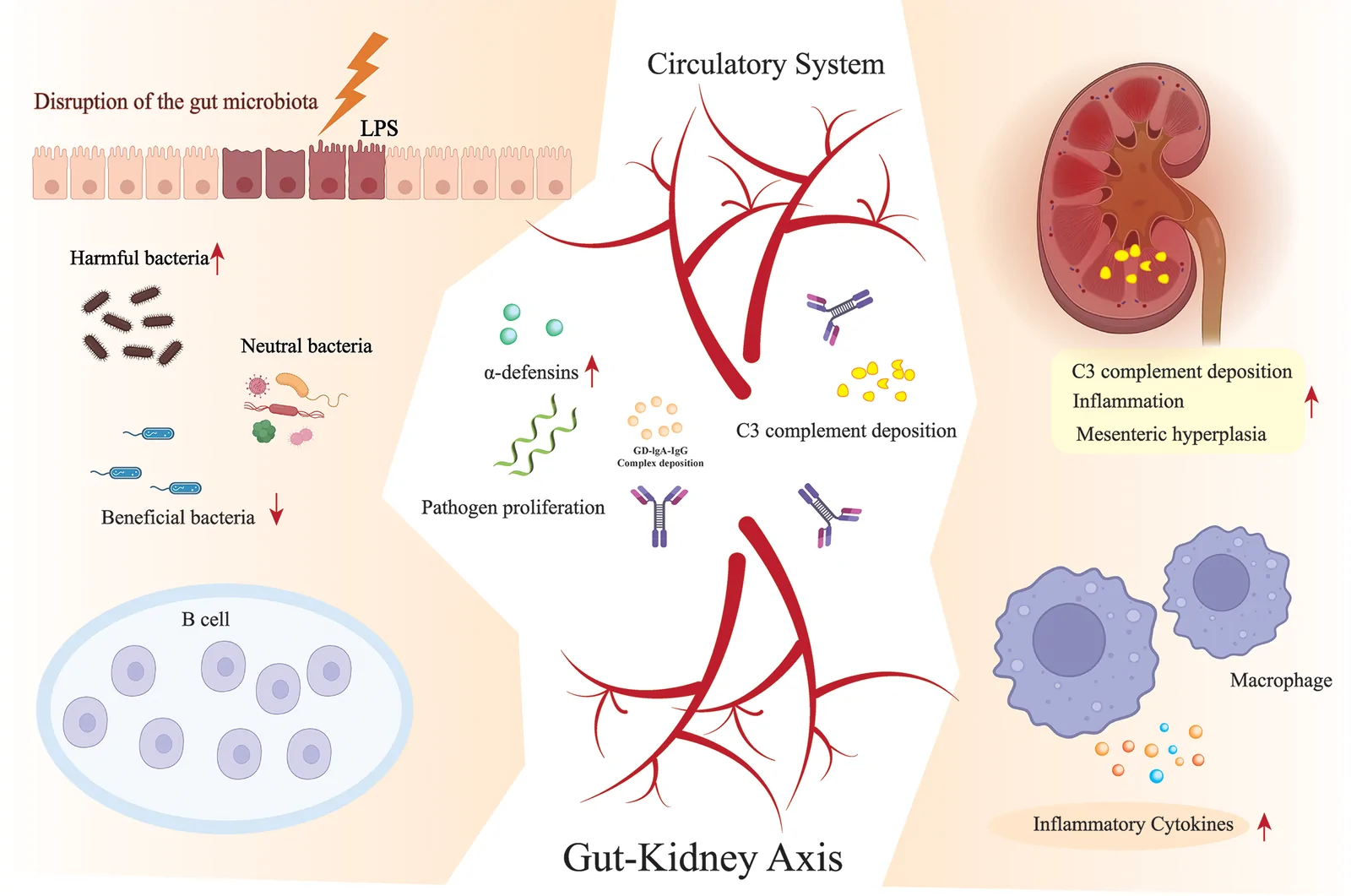
Imbalance of the gut–kidney axis in IgAN. This schematic summarizes the proposed “vicious cycle” that links intestinal barrier disruption, mucosal immune activation, and renal immune-complex injury in IgAN. The diagram illustrates the bidirectional cross talk between the gastrointestinal tract and the kidneys in this disease. The intestinal barrier comprises a monolayer of columnar epithelial cells interspersed with goblet cells that secrete a protective mucus layer. Damage to the epithelial barrier increases permeability, allowing microbial products such as lipopolysaccharide (LPS) to translocate across the mucosa. The gut microbiota consists of pathogenic, beneficial, and neutral commensal bacteria, with dysbiosis—characterized by compositional imbalance—commonly observed in IgAN. Gut dysbiosis promotes mucosal immune activation and enhances Gd-IgA1 synthesis. Peyer’s patches, which harbor IgA1-secreting B cells, serve as the principal sites of IgA production within the intestinal mucosal immune system and represent critical hubs for the aberrant generation of Gd-IgA1. In the circulation, Gd-IgA1 is recognized by anti-glycan autoantibodies (e.g., IgG), leading to the formation of IgA–IgG ICs. This process is accompanied by reduced α-defensin expression and complement C3 activation, which drive the expansion of pathogenic immune aggregates and amplify inflammatory signaling. Within the kidneys, circulating Gd-IgA1–IgG complexes and complement C3 deposit in the glomerular mesangium, triggering mesangial cell proliferation, glomerulosclerosis, and macrophage-mediated release of pro-inflammatory cytokines. These events provoke inflammation and proteinuria, thereby exacerbating renal injury. The accumulation of uremic toxins reflects diminished renal clearance capacity. These metabolites have been shown to further disrupt epithelial tight junction integrity and alter gut microbial composition, thereby perpetuating the pathogenic cycle. This scheme exemplifies the “bidirectional communication” inherent to the gut–kidney axis: intestinal perturbations affect the kidneys via metabolites and immune mediators, while renal impairment, in turn, worsens the gut environment. This reciprocal interaction establishes a self-sustaining pathological loop that underlies the dysregulation of the gut–kidney axis in IgAN.

The gut–kidney axis constitutes a reciprocal system through which the intestine and kidneys communicate via bidirectional signaling pathways. Its core components involve the dynamic cross talk among gut microbes and their metabolites, intestinal barrier integrity, immune regulatory networks, and renal function (33, 35). Disruption of the intestinal microbiota can impair barrier integrity, allowing uremic toxins—such as indoxyl sulfate (IS)—to enter the circulation, thereby triggering systemic inflammation and accelerating the decline in renal function (20, 33, 36–38). The uremic environment arising from kidney diseases, including CKD, can further aggravate gut microbial dysbiosis and compromise intestinal barrier function, thus establishing a vicious cycle of pathological alterations (33, 39, 40). A substantial body of evidence has established that this bidirectional regulatory axis plays a critical role in the pathogenesis and progression of various kidney disorders, including IgAN as depicted in **Figure 2** (3, 22, 41).

### 2.2 Components of the gut–kidney axis

#### 2.2.1 Gut microbiota

The gut microbiota in IgAN patients exhibits marked dysbiosis, characterized by increased abundance of genera such as *Streptococcus* and *Escherichia-Shigella*, alongside reduced levels of *Faecalibacterium*, *Roseburia*, and *Bifidobacterium* (42). Through their metabolic products, gut microbes can modulate intestinal immune responses and, in turn, affect the glycosylation pattern of IgA. Emerging evidence indicates that microbial dysbiosis may play a substantial role in driving the overproduction of Gd-IgA1, which is considered the key pathogenic factor in IgAN development (10, 43, 44).

#### 2.2.2 Intestinal barrier

The intestinal barrier is a multilayered structure composed of the mucus layer, intestinal epithelial cells, and intercellular tight junction proteins (45). Its primary function is to maintain homeostasis at the interface between the gut microbiota and the host. In IgAN patients, intestinal permeability is abnormally increased, which facilitates the translocation of microbial metabolites—including lipopolysaccharide (LPS)—across the epithelial barrier and into the circulation. Breach of the intestinal barrier exposes bacterial antigens to the systemic circulation, triggering a systemic immune response and ultimately promoting glomerular IgA deposition (10, 46).

#### 2.2.3 Immune system

The immune system serves as a central mediator within the gut–kidney axis, operating through two interrelated compartments—mucosal immunity and systemic immunity. From the mucosal perspective, Peyer’s patches in the terminal ileum represent the primary sites of IgA production (47). Disruption of the intestinal microbial equilibrium, leading to dysbiosis, activates mucosal B cells and drives the overproduction of Gd-IgA1 (3). These excess Gd-IgA1 molecules subsequently form ICs that deposit in renal tissue, thereby causing kidney injury. At the systemic level, TLR4 recognizes pathogen-associated molecular patterns (PAMPs) derived from the gut microbiota. Such recognition triggers the release of pro-inflammatory mediators, which further exacerbate renal injury (13, 48).

#### 2.2.4 Kidneys

Renal function and gastrointestinal health are closely interrelated. The kidneys, in turn, exert a reciprocal influence on the gut. Progressive decline in renal function leads to systemic accumulation of uremic toxins, which compromise intestinal barrier integrity, exacerbate microbial dysbiosis, and thereby perpetuate a vicious cycle (38, 49). In the specific context of IgAN, impaired renal clearance allows circulating Gd-IgA1–containing ICs to activate the complement system, which in turn accelerates the development of glomerulosclerosis (50).

### 2.3 Physiological mechanisms of the gut–kidney axis

#### 2.3.1 Metabolic regulation

SCFAs, produced by gut microbial fermentation, regulate host metabolism and immune responses through G protein–coupled receptors and also help maintain epithelial barrier integrity (51, 52). In population-based studies, reduced fecal SCFA levels in IgAN patients correlate with microbial dysbiosis and clinical disease phenotypes, suggesting that impaired anti-inflammatory signaling may contribute to exacerbated renal inflammation (53). Furthermore, microbiota-derived indole derivatives—which include sulfated indolic uremic toxins—are tryptophan metabolites that act as ligands for the aryl hydrocarbon receptor (AhR) and can enhance inflammatory responses in an AhR-dependent manner. These compounds induce cellular senescence and fibrosis in renal tubular epithelial cells, thereby promoting tubulointerstitial remodeling (54–56). Tryptophan catabolites derived from the microbiota—including indole and various indole derivatives—represent a major category of host–microbe co-metabolites that possess immunomodulatory properties. Indolic uremic toxins, such as IS, are produced when bacterially generated indole intermediates undergo further modification by host hepatic sulfotransferases (57). These ligands can activate AhR, a receptor widely expressed in the intestinal microenvironment—including epithelial and immune cells—and associated with barrier-protective pathways (58). Downstream of AhR activation, signaling intersects with mucosal defense mechanisms, including IL-22–dependent epithelial protection and antimicrobial responses, which in turn can modulate the composition of the gut microbiota (59). Concurrently, IS has been demonstrated to promote pro-inflammatory innate immune reprogramming—for instance, trained immunity in monocytes—via AhR-dependent pathways, offering a plausible mechanism for amplifying systemic inflammation in the setting of kidney disease (54).

#### 2.3.2 Immune regulation

##### 2.3.2.1 TLR4 signaling pathway

The Toll-like receptor (TLR) signaling pathway represents a central molecular nexus that connects gut dysbiosis to the overproduction of galactose-deficient IgA1 (Gd-IgA1). Intestinal-derived LPS, a canonical ligand for TLR4, engages TLR4 and subsequently recruits myeloid differentiation factor 88 (MyD88), leading to activation of the NF-κB cascade.

*In-vitro* work by Zhu et al. demonstrated that B cells derived from peripheral blood mononuclear cells of patients with IgAN exhibited marked elevations in MyD88 and NF-κB phosphorylation following LPS challenge, together with increased expression of BAFF (B-cell activating factor) and APRIL (a proliferation-inducing ligand) (22). Notably, these responses were substantially attenuated by administration of the TLR4-selective inhibitor TAK-242. This discovery indicates that the TLR4/MyD88/NF-κB axis is the key signaling pathway through which dysbiosis promotes the production of Gd-IgA1.

In addition to TLR4, other Toll-like receptor pathways have also been implicated in IgAN pathogenesis, as discussed below.

##### 2.3.2.2 TLR9 signaling pathway

Toll-like receptor 9 (TLR9), in contrast, is an intracellular sensor that recognizes unmethylated CpG motifs present in bacterial and viral DNA. Makita et al. demonstrated that stimulation of tonsillar mononuclear cells from IgAN patients with TLR9 agonists (CpG oligonucleotides) led to marked upregulation of APRIL and IL-6, which in turn induced B cells to produce galactose-deficient IgA1 (Gd-IgA1); this effect was abrogated by a TLR9-specific inhibitor or by neutralizing antibodies against APRIL (60). These findings further confirm that TLR9 signaling contributes to IgAN pathogenesis via the TLR9–APRIL/IL-6–Gd-IgA1 axis. At the *in-vivo* level, research by Fukao et al. showed that plasmacytoid dendritic cells (pDCs) in the tonsils of IgAN patients correlated positively with TLR9 and APRIL expression and that TLR9-positive pDCs could drive the production of Gd-IgA1 (61). Thus, both TLR4 and TLR9 pathways converge on the upregulation of BAFF/APRIL signaling, which represents a central hub in Gd-IgA1 production.

##### 2.3.2.3 The BAFF/APRIL signaling pathway

The BAFF/APRIL signaling pathway serves as a molecular bridge that links signals derived from the microbiota to the production of Gd-IgA1. BAFF and APRIL belong to the tumor necrosis factor superfamily. They are critical regulators of B-cell survival, differentiation, and immunoglobulin class switching (48, 62). In patients with IgAN, serum concentrations of BAFF and APRIL are markedly elevated and correlate positively with Gd-IgA1 levels as well as disease severity. A systematic review by Cheung et al. clearly shows that BAFF and APRIL function through binding to three receptors on the B-cell surface, namely BAFF-R, TACI, and BCMA (48). In particular, BAFF primarily supports B-cell survival and homeostasis, whereas APRIL preferentially drives immunoglobulin class switching and plasma cell differentiation.

Within the mucosal immune environment, APRIL plays a particularly critical role. Dendritic cells in intestinal Peyer’s patches secrete APRIL upon sensing microbial signals, particularly TLR9 ligands, and this in turn induces T-cell–independent IgA class switching in B cells (63). The pathological relevance of this mechanism in IgAN is that intestinal dysbiosis leads to continuous exposure of dendritic cells to abnormally elevated microbial ligands, which results in overproduction of APRIL and subsequently drives B cells to generate large quantities of aberrantly glycosylated IgA. Gao et al. observed that serum levels of Gd-IgA1-positive bacterial-specific antibodies were significantly elevated in IgAN patients and correlated positively with serum APRIL concentrations (43). These findings further support the pathogenic sequence from microbiota dysbiosis to increased APRIL and ultimately to excessive Gd-IgA1 production.

On the therapeutic front, agents targeting the BAFF/APRIL pathway have entered clinical trials and demonstrated considerable efficacy. Sibeprenlimab, an anti-APRIL monoclonal antibody, reduced urinary protein in patients during a Phase II trial, along with a decrease in serum Gd-IgA1 level (64). Atacicept, a TACI-Fc fusion protein that simultaneously blocks both BAFF and APRIL, also showed renal protective effects in a Phase IIb trial (65). Collectively, these clinical data provide reverse evidence from a therapeutic standpoint, confirming the central role of the BAFF/APRIL pathway in IgAN pathogenesis. Beyond B-cell activation, complement system activation represents another key effector pathway downstream of immune complex deposition.

##### 2.3.2.4 Complement activation pathway

Following deposition of ICs in the glomerular mesangium, they predominantly activate the complement system via both the alternative and lectin pathways, leading to glomerular injury (9, 66). Immunohistochemical analyses of renal biopsy specimens from IgAN patients have identified proteins associated with the alternative pathway, including resolvins, complement factor B, factor H, CFHR1, and CFHR5, as well as lectin pathway proteins such as mannose-binding lectin, MBL-associated serine proteases 1/2, and C4 (67).

The alternative pathway is regarded as the dominant route of complement activation in IgAN. Chiu et al. reported that serum alternative pathway activity in IgAN patients correlated positively with Gd-IgA1 antibody levels and also with the degree of proteinuria (67). On the clinical trial front, C3 inhibitors and factor B inhibitors that target the alternative pathway have entered clinical development. Iptacopan, an oral factor B inhibitor, significantly reduced proteinuria in IgAN patients in a Phase III trial and received breakthrough therapy designation from the US FDA (68).

Lectin pathway activation is also associated with more severe disease progression. Medjeral-Thomas et al. pointed out that MBL deposition in the glomerular mesangium, in the context of IgA ICs, is an independent predictor of poor prognosis in IgAN. At present, inhibitors targeting the lectin pathway are also in preclinical and early stage clinical development (9). Collectively, the TLR4, TLR9, BAFF/APRIL, and complement pathways form an integrated signaling network that links microbial stimuli to renal injury.

##### 2.3.2.5 Synergistic interaction between TLR4 and TLR9 and the complement-bacterial community axis

Notably, the TLR4 and TLR9 signaling pathways may act synergistically in the pathogenesis of IgAN. Intestinal LPS (a TLR4 ligand) and CpG DNA from upper respiratory tract pathogens (a TLR9 ligand) can each activate systemic B-cell responses, thereby jointly driving excessive Gd-IgA1 production. This mechanism also accounts for why infections—particularly those of the intestine and upper respiratory tract—are common triggers for the onset and exacerbation of IgAN.

Furthermore, there may be a direct link between complement activation and intestinal microbiota imbalance. Research by Zhu et al. demonstrated that mice transplanted with microbiota from IgAN patients exhibited increased glomerular C3 deposition, suggesting that the gut microbiota might influence the activation threshold of the complement system via certain mechanisms (22). This axis linking the microbiota and complement awaits further investigation.

In summary, the immune regulatory mechanisms described above constitute a multilayered network connecting gut microbiota dysbiosis to Gd-IgA1 production and subsequent renal injury. TLR4 and TLR9 serve as proximal sensors of microbial products, BAFF and APRIL act as central amplifiers of B-cell responses, and the complement system functions as a distal effector of tissue damage. These pathways do not operate in isolation; rather, they interact synergistically to amplify pathogenic signals (22, 60). Understanding the integration of these pathways is critical for identifying optimal therapeutic targets within the gut–kidney axis.

#### 2.3.3 Maintenance of barrier function

Lactobacillus and other commensal bacteria can enhance the expression of mucins and tight junction proteins to preserve the integrity of the intestinal barrier (69, 70). The presence of microecological imbalance in patients with IgAN, coupled with compromised barrier function, indicates an elevated risk of endotoxin translocation (22). At the intervention level, animal and preliminary translational studies indicate that probiotics, primarily consisting of Lactobacillus or Bifidobacterium, can ameliorate microbiota and barrier function while diminishing IgA deposition and histological damage (28). However, conclusive clinical evidence confirming that “probiotics significantly lower serum Gd-IgA1 levels in patients” is currently absent. Conversely, budesonide, which targets the ileal mucosa, has been shown in population studies to reduce Gd-IgA1 and associated ICs (71).

### 2.4 Genetic susceptibility contributes to IgAN onset via the mucosal barrier, microbial sensing, and immune effector pathways

The genome-wide association study (GWAS) loci discussed above do not operate in isolation; rather, they together constitute a pathogenic network that exhibits a stepwise, hierarchical organization. In this section, we organize these loci into four functional tiers summarized in **Table 1** to illustrate how genetic variants drive Gd‑IgA1 production and subsequent renal deposition through the gut–kidney axis.

**Table 1:** Genetic risk loci identified by GWAS in IgAN and their proposed functional roles.

IgAN risk loci	Main biological functions	The mechanisms and evidence related to IgAN
*HLA*	Mucosal surface antigen presentation and adaptive immunity	It affects mucosal antigen processing and downstream IgA response, determining susceptible subtypes (72)
*DEFA*	Production of Pan’s cell antimicrobial peptides (defensins); Regulate the composition of the microbiota and the integrity of the barrier	Reducing the antibacterial defense capacity can easily lead to flora imbalance and barrier damage. The GWAS signal is located at this site (72, 73)
*CARD9*	Innate immune aptamer protein; Regulating intestinal inflammation and host-microbe balance	It affects the production of AhR ligands by tryptophan metabolism in microorganisms and weakens the barrier protection mediated by IL-22 (74); Associated with dysbiosis in IgAN (72)
*ITGAM/ITGAX*	Microbial identification and clearance of myeloid cells	It affects the function of mucosal myeloid cells and indirectly alters the composition of the microbiota and barrier inflammation (72, 75)
*TNFSF13(APRIL)*	B-cell survival and Ig category conversion	It plays a key role in the category conversion of mucosal IgA; Genetic variations can increase the production of pathogenic IgA (62, 72)
*TNFRSF13B (TACI)*	BAFF/APRIL receptor; B cell signaling and IgA regulation	Regulates B cell survival and class switching downstream of microbial signals, and affects the quantity and quality of IgA (48, 72)
*FCAR (CD89)*	The effector function of IgA ICs	It affects the processing of IgA ICs and the amplification of inflammation. Once pathogenic IgA forms, it can aggravate renal injury (72, 76)
*IGH*	The genetic structure of antibody production (including IgA)	It affects the production capacity of IgA and interacts with mucosal antigen stimulation (72)

#### 2.4.1 First layer: mucosal barrier and microbial defense (*DEFA* and *CARD9*)

Paneth cells are specialized epithelial cells situated at the base of intestinal crypts. They help regulate the composition of the gut microbiota and preserve barrier integrity through the secretion of antimicrobial peptides, including α-defensins (77). The *DEFA* risk locus, as identified by GWAS in IgAN, maps to the defensin gene cluster region, implying that genetic variants contributing to impaired Paneth cell function may reduce local antibacterial activity. This reduction could in turn permit overgrowth of opportunistic pathogens, particularly mucin-degrading bacteria such as *Akkermansia muciniphila* (AKK) (72). In parallel, *CARD9* serves as a critical adaptor protein that governs intestinal fungal recognition and antifungal immune responses. Lamas et al. showed in a mouse model that *CARD9* knockout impairs the capacity of the gut microbiota to metabolize tryptophan into AhR ligands, consequently attenuating the IL-22–mediated barrier protective response (74). In patients with IgAN, the risk allele at the *CARD9* locus may aggravate intestinal barrier dysfunction and microbial dysbiosis via a comparable mechanism (72).

#### 2.4.2 Second layer: microbial sensing and signal amplification (indirectly associated with *CARD9*, *ITGAM*, and TLR pathways)

When the mucosal barrier is compromised, microbes or microbial products, including LPS and CpG DNA, may translocate to the submucosal tissues, where innate immune cells detect these stimuli via pattern recognition receptors. *CARD9* is not only involved in antifungal defense but also contributes to modulating the threshold of inflammatory responses in myeloid cells upon exposure to diverse microbial ligands (78). *ITGAM*, which encodes CD11b, constitutes the integrin αM chain and is essential for myeloid cell chemotaxis and phagocytosis. Sim et al. observed that Cd11b-deficient mice displayed exaggerated inflammatory responses in infection models (75). In the setting of IgAN, the risk variant at *ITGAM* may predispose myeloid cells to hyperresponsiveness toward intestinal microbial ligands, thereby amplifying the activation of TLR4 and TLR9 signaling pathways (22, 60, 72, 79).

#### 2.4.3 Third layer: B-cell activation and IgA class switching (*TNFSF13*/APRIL and *TNFRSF13B*/TACI)

Signals arising from the gut microbiota are continuously relayed to B cells through APRIL and BAFF. The GWAS-identified risk loci at *TNFSF13*, which encodes APRIL, and *TNFRSF13B*, which encodes TACI (the receptor for both APRIL and BAFF), directly impinge on the downstream components of this signaling axis (72). Individuals who carry these risk alleles may display enhanced sensitivity to APRIL signals originating from the microbiota, such that B cells undergo class switching and differentiate into IgA-secreting plasma cells even at reduced levels of stimulation. This hyper-reactive state closely parallels the overproduction of Gd-IgA1 that is characteristically observed in patients with IgAN (48, 62).

#### 2.4.4 Fourth layer: immune complex processing and kidney injury (*FCAR* and *HLA*)

After Gd-IgA1 enters the circulation and forms ICs with anti-glycan autoantibodies, its clearance rate and propensity for tissue deposition are influenced by the *FCAR* gene (encoding FcαRI/CD89) and *HLA* genotypes (72, 76). FcαRI serves as the principal receptor for IgA ICs and mediates endocytosis and clearance of these complexes on myeloid cell surfaces. *HLA* molecules participate in antigen presentation and may affect the generation of autoantibodies directed against Gd-IgA1. Functional impairment of FcαRI or particular *HLA* subtypes may result in delayed immune complex clearance, enhanced glomerular deposition, and excessive complement activation.

This hierarchical framework integrates the originally independent GWAS loci into a causal chain from the gut to the kidneys: genetically determined barrier vulnerability (*DEFA* and *CARD9*) leads to dysbiosis and aberrant microbial signal exposure, which in turn triggers myeloid cell hyperresponsiveness (*CARD9* and *ITGAM*), followed by APRIL/BAFF-driven overactivation of B cells (*TNFSF13* and *TNFRSF13B*), resulting in excessive Gd-IgA1 production and immune complex deposition, and finally FcαRI/*HLA*-determined susceptibility to renal injury (*FCAR* and *HLA*). This framework not only accounts for the genetic heterogeneity of IgAN but also offers a theoretical foundation for precision therapies directed at specific nodes, such as APRIL and TLR4.

## 3 Gut microbiota and IgAN

### 3.1 Causal relationship and bidirectional interaction between gut microbiota dysbiosis and IgAN

The causal link between gut microbiota and IgAN remains a subject of debate. In this section, we draw on evidence from Mendelian randomization (MR) studies, animal models, and clinical cohorts to systematically discuss the bidirectional interaction between the two, following a progressive framework of driving, reshaping, and cycling.

#### 3.1.1 Gut microbial dysbiosis as a potential driver of IgAN: causal evidence

To address the question of whether gut dysbiosis is a cause or a consequence of IgAN, we first examine the evidence for a causal role of the microbiota. Several large-scale two-sample MR studies have leveraged genome-wide association data to evaluate whether specific gut microbiota taxa are causally linked to IgAN risk. Wang et al. used 211 gut microbial groups as instrumental variables and confirmed that gut dysbiosis directly elevates the risk of developing IgAN (26). Among these, the phylum Actinobacteria was the only taxon that remained significant across multiple testing corrections, and its abundance correlated significantly with proteinuria levels and clinical prognosis in patient cohorts. Ren et al. further corroborated this causal direction and ruled out the influence of reverse causation (25). Wan et al. further expanded these findings through an MR analysis of two groups of samples, specifically studying the mediating role of immune cell subgroups (80). Their research confirmed that specific B-cell and T-cell subgroups play a mediating role in the path from intestinal flora imbalance to the onset of IgAN, providing more refined genetic evidence for the one-way causal chain where microbial population changes increase the risk of IgAN.

In animal studies, humanized microbiota transplantation models have provided strong supportive evidence for the pathogenic role of gut microbes. Zhu et al. transferred fecal microbiota from IgAN patients or healthy controls into germ-free mice (22). They observed that mice receiving patient-derived microbiota exhibited significantly elevated serum Gd-IgA1 levels, increased glomerular IgA and C3 deposition, downregulation of intestinal barrier proteins such as ZO-1 and occludin, and activation of both the TLR4/MyD88/NF-κB and BAFF/APRIL signaling pathways. Lauriero et al. reached similar conclusions using humanized IgAN transgenic mice (α1KI-CD89Tg) (24). Following transplantation with IgAN patient microbiota, these mice showed increased urinary albumin excretion, along with mesangial expansion, IgA1 deposition, and inflammatory cell infiltration in renal tissue. Collectively, these lines of evidence support that gut microbial dysbiosis acts as an independent driving factor in the pathogenesis of IgAN.

#### 3.1.2 Reverse remodeling of the gut microbiota by the pathological state of IgAN: disease-driven microbiota alterations

Conversely, emerging evidence suggests that the disease state of IgAN may itself reshape the gut microbiota, indicating a bidirectional relationship. In animal models, humanized IgAN mice, such as the α1KI-CD89Tg strain, even without receiving patient microbiota transplants, display reduced microbial diversity and enrichment of certain pathogenic taxa, including *Escherichia-Shigella*, under specific conditions (24, 81). In human studies, multiple cross-sectional investigations and meta-analyses have consistently reported decreased gut microbial alpha diversity in IgAN patients, along with an increased abundance of *Bacteroides* and *Escherichia-Shigella*, as well as a reduced abundance of short-chain fatty acid-producing bacteria such as *Faecalibacterium* and *Ruminococcus* (42, 82, 83). Cai et al. further found that this microbial disturbance is not confined to the gut but also involves the oral and pharyngeal compartments, implying that IgAN represents a systemic disorder of mucosal microbiota homeostasis affecting multiple sites (84).

From a pathophysiological standpoint, the decline in renal function attributable to IgAN results in the accumulation of urea and uremic toxins, including IS and p-cresyl sulfate, in the systemic circulation. These toxic metabolites can disrupt intestinal epithelial tight junctions, provoke oxidative stress and inflammatory reactions, and subsequently aggravate microbial dysregulation while compromising gut barrier integrity (38, 85). Thus, the disease state itself can both promote and sustain microbial dysregulation via a kidney–gut feedback loop.

To systematically summarize the principal alterations in the gut microbiota of IgAN patients across the phylum, genus, and species levels, along with their mechanistic links to Gd-IgA1 production and intestinal barrier integrity, we have compiled the consistently reported microbial changes described above in **Table 2**.

**Table 2:** Altered gut microbial taxa in IgAN and their mechanistic links to Gd‑IgA1 production and intestinal barrier function.

Taxon (rank)	Direction in IgAN	Associated with the mechanisms of Gd-IgA1 production and/or intestinal barrier dysfunction	Key metabolites/PAMPs/enzymes	Host immune pathways	References
*Proteobacteria* (phylum)	↑	Both (barrier disruption + Gd-IgA1 via TLR4-BAFF/APRIL)	LPS; flagellin	TLR4/MyD88/NF-κB → B-cell stimulators (BAFF/APRIL)	(42)
*Streptococcus* (genus)	↑	Barrier disruption (putative Gd-IgA1)	Cell wall components (LTA, peptidoglycan)	TLR2/MyD88 (putative);	(42)
*Paraprevotella* (genus)	↑	Barrier disruption (LPS-induced leaky gut)	LPS	TLR4/MyD88/NF-κB	(42)
*Escherichia–Shigella* (genus)	↑	Both (barrier disruption + Gd-IgA1 via BAFF/APRIL)	LPS; flagellin	TLR4/MyD88/NF-κB → BAFF/APRI	(22, 42)
*Akkermansia muciniphila* (species)	↑	Gd-IgA1 generation (IgA1 deglycosylation)	Mucin-degrading enzymes (glycosidases, proteases)	IgA1 deglycosylation → Gd-IgA1 neoepitope formation → ICs	(10)
*Fusicatenibact*er (genus)	↓	Barrier protection (SCFA-mediated)	SCFAs (butyrate)	SCFA–GPCR signaling (GPR41/GPR43/GPR109A) → barrier integrity	(42, 86, 87)
*Roseburia* (genus)	↓	Barrier protection (SCFA-mediated)	SCFAs (butyrate)	SCFA–GPCR signaling (GPR41/GPR43/GPR109A) → barrier integrity	(86, 87)
*Ruminococcaceae* (family)	↓	Barrier protection (SCFA-mediated)	SCFAs (butyrate and others)	SCFA–GPCR signaling (GPR41/GPR43/GPR109A) → barrier integrity	(42, 86, 87)
*Lachnospiraceae* (family)	↓	Barrier protection (SCFA-mediated)	SCFAs (butyrate/propionate/acetate)	SCFA–GPCR signaling (GPR41/GPR43/GPR109A) → barrier integrity	(42, 86, 87)

↑indicates enrichment/increase in IgAN, ↓indicates decrease in IgAN.

#### 3.1.3 Positive feedback cycle of the gut–kidney axis: consolidation and amplification of the bidirectional relationship

Building on the evidence presented above, we propose a self-perpetuating positive feedback loop that integrates both directions of causality. The sequence of events is as follows:

1. Initial phase: genetic predisposition (such as variants at the *DEFA* and *CARD9* loci) or environmental triggers (including diet and infections) give rise to gut microbial dysbiosis and mucosal barrier injury (44, 72).
2. Amplification phase: dysbiosis activates the TLR4/TLR9 signaling axis and the BAFF/APRIL pathway, which drives B cells to generate substantial quantities of Gd-IgA1. Concurrently, the compromised barrier permits endotoxins (e.g., LPS) and uremic toxin precursors to gain access to the bloodstream (22, 60).
3. Effector phase: Gd-IgA1 forms ICs with autoantibodies and deposits within the glomeruli, which activate the complement system (via both the alternative and lectin pathways) and recruit inflammatory cells, ultimately resulting in glomerulosclerosis and a progressive loss of renal function (9, 67).
4. Feedback phase: declining renal function further impairs toxin clearance and elevates circulating levels of uremic toxins, such as IS. These toxic metabolites, in turn, injure the intestinal barrier and exacerbate microbial dysbiosis, thereby completing a reverse reinforcing loop from the kidney back to the gut (33, 85).

This positive feedback cycle accounts for the frequently observed chronic progressive worsening of IgAN once it reaches the clinical stage and offers a theoretical rationale for combined therapeutic approaches that target both the gut and the kidneys simultaneously.

### 3.2 Role of gut microbiota metabolites in IgAN

The gut microbiota influences host immune function and renal health through the production of a wide range of metabolites. In this section, we outline these mechanisms according to the major metabolite classes.

#### 3.2.1 SCFAs

SCFAs, primarily acetate, propionate, and butyrate, are the principal metabolites generated from gut bacterial fermentation of dietary fiber. Their fecal concentrations are significantly lower in patients with IgAN (53). SCFAs exert anti-inflammatory and barrier-protective effects through binding to G protein–coupled receptors, including GPR41, GPR43, and GPR109A. They promote regulatory T-cell differentiation and suppress Th17 responses, while also enhancing the expression of intestinal epithelial tight junction proteins and reducing the translocation of pro-inflammatory molecules such as LPS (51, 86). In animal models of IgAN, supplementation with SCFAs attenuates glomerular IgA deposition and mesangial proliferation (28).

#### 3.2.2 Tryptophan metabolites and AhRs

The gut microbiota metabolizes dietary tryptophan into indole and its derivatives, such as indole-3-acetic acid, indole-3-propionic acid, and IS, among others. These metabolites serve as important ligands for AhRs (58). AhR, a member of the basic helix-loop-helix transcription factor family, is widely expressed in intestinal epithelial cells, immune cells, and renal cells. Under healthy conditions, AhR signaling helps maintain intestinal barrier integrity and induces IL-22–mediated mucosal protection (59). However, in the context of CKD, uremic toxins such as IS can induce a trained immunity phenotype in monocytes through the AhR-dependent pathway, promoting the release of inflammatory factors (54). Concurrently, they activate the AhR-NLRP3 inflammasome pathway in renal tubular epithelial cells, thereby accelerating renal fibrosis (54, 55). Wu et al. found that the serum tryptophan metabolite profile of IgAN patients showed significant alterations, which were associated with disease severity (88). Zhang et al. further confirmed through a multi-omics study that levels of tryptophan derivatives in feces and serum can effectively distinguish IgAN patients from healthy controls (89).

#### 3.2.3 Uremic toxins: IS, p-cresyl sulfate, and related compounds

IS and p-cresyl sulfate are typical examples of protein-bound uremic toxins. Their precursors, indole and p-cresol, are generated by the gut microbiota from aromatic amino acid metabolism and undergo sulfation in the liver prior to entering the circulation (57). As renal function declines, the clearance of these toxins diminishes and their serum concentrations rise (90). Experimental evidence demonstrates that IS directly injures renal tubular epithelial cells and induces epithelial-mesenchymal transition; promotes inflammation and oxidative stress through activation of the AhR and NF-κB pathways; and disrupts intestinal tight junctions, thereby establishing a vicious cycle (91, 92). Hou et al. confirmed in sulfotransferase 1a1 knockout mice that reducing IS accumulation alleviates renal fibrosis (91).

#### 3.2.4 Bile acids and other metabolites

Bile acids are transformed into secondary bile acids, including deoxycholic acid and cholic acid, via dissociation and dehydroxylation mediated by gut microbes. Their metabolism is further governed by the Farnesoid X receptor and the G-protein-coupled bile acid receptor 1 (93). Patients with IgAN also exhibit an aberrant serum bile acid profile (89). In addition, trimethylamine oxide, which is generated from microbial metabolism of choline and carnitine, may aggravate renal inflammation and fibrosis through activation of the NLRP3 inflammasome and promotion of macrophage infiltration (94). At present, direct evidence for the pathogenic involvement of these metabolites in IgAN remains limited, and further investigation is needed.

#### 3.2.5 Emerging microbial metabolites identified in recent studies

Recent studies published in 2026 have uncovered previously unrecognized microbial metabolites potentially involved in IgAN pathogenesis. One integrated analysis of gut metagenomics and glomerular spatial transcriptomics revealed that IgAN patients exhibit enrichment of acetate metabolism pathways in their gut microbiome, accompanied by elevated systemic acetate levels and altered expression of metabolic and signaling genes within glomeruli (95). Another study identified that the gut microbial metabolite sphingosine-1-phosphate (S1P) can upregulate CCL2 expression in renal mesangial cells, activating MET/FAK signaling and promoting mesangial cell proliferation, thereby accelerating IgAN progression (96). In addition, multi-omics analyses of gut microbiota and metabolome remodeling during the early post-transplant period in IgAN patients with ESRD have revealed important roles for microbial metabolites in transplant recovery. These emerging findings expand our understanding of how microbial metabolites contribute to IgAN pathogenesis and point toward novel therapeutic strategies targeting specific metabolites.

### 3.3 Role of oral and respiratory tract microbiota in IgAN

Accumulating evidence indicates that IgAN is not confined to the gut but rather represents a systemic mucosal immune disease marked by microbial dysbiosis at multiple mucosal surfaces. In addition to intestinal alterations, patients with IgAN show compositional shifts in the oral and upper respiratory microbiota, characterized by an expansion of pro-inflammatory taxa and a decline in commensal species critical for maintaining mucosal homeostasis (1, 82). Clinically, the frequent observation that IgAN onset or relapse occurs shortly after tonsillitis, upper respiratory tract infections, or oral inflammatory conditions further supports a role for extraintestinal microbiota in modulating disease activity (1).

Recent multi-niche profiling has revealed that IgAN is associated with microbial dysbiosis extending beyond the intestinal tract. In a large-scale 16S rRNA sequencing study covering oral, pharyngeal, gut, and urine niches, IgAN patients exhibited niche-specific enrichment of opportunistic taxa—such as *Bergeyella* and *Capnocytophaga*—within the oral and pharyngeal communities (84). The abundance of these taxa in oral and pharyngeal samples correlated positively with serum creatinine and urea levels, suggesting a potential link between oropharyngeal dysbiosis and renal impairment.

Alterations in the tonsillar microbiota may also contribute to disease modulation. Comparative analyses of tonsillar microbiota have revealed genus-level compositional differences between IgAN patients, patients with other renal diseases, and healthy controls (97). These findings reinforce the view that the tonsil serves not only as a clinical trigger site but also as a potential mucosal reservoir relevant to IgAN pathogenesis.

At the molecular level, one plausible mechanism is that increased exposure to microbial ligands and inflammatory mediators at oral and upper airway mucosal surfaces amplifies systemic innate immune activation, thereby promoting B-cell stimulation and enhancing Gd-IgA1 production. In IgAN, LPS can activate the TLR4/MyD88/NF-κB axis in peripheral blood mononuclear cells, driving the expression of B-cell stimulatory factors and pro-inflammatory cytokines. *In-vitro* studies have shown that this process is associated with increased Gd-IgA1 production and can be attenuated by TLR4 inhibition (22). In parallel, TLR9-driven innate signaling has been reported to enhance the production of aberrantly glycosylated IgA via APRIL and interleukin-6 pathways, offering a mechanistic basis for infection-associated disease flares at upper airway mucosal sites (60). Beyond systemic innate signaling, upper airway lymphoid tissues may directly influence IgA glycosylation programs. IgA1-producing cell lines derived from the tonsils of IgAN patients show enhanced Gd-IgA1 production under LIF2-STAT1 signaling, supporting a direct link between cytokine signaling and aberrant glycosylation within the tonsillar immune microenvironment (98).

Based on the correlational evidence summarized above, a working model of an “oropharyngeal mucosa–kidney axis” has been proposed, in which oral and pharyngeal dysbiosis may heighten mucosal immune activation and systemic inflammatory tone, thereby potentially lowering the threshold for pathogenic IgA responses and acute immune-complex–mediated renal injury (1). This model, however, remains hypothetical and awaits validation through longitudinal sampling and mechanistic studies in humans. Biologically, cross talk between these mucosal sites is plausible, as the oral–respiratory–gut continuum constitutes an interconnected immunological network. Microbial communities residing in the oral cavity can translocate to the gastrointestinal tract via swallowing, thereby influencing gut microbial composition and local immune tone. Conversely, intestinal dysbiosis may modulate systemic IgA responses and reshape mucosal immunity at distant sites, including the oropharynx (99). Shared immunological pathways—including TLR-mediated activation, BAFF/APRIL signaling, and mucosal dendritic cell conditioning—may integrate microbial signals from distinct mucosal surfaces and collectively drive the overproduction of Gd-IgA1.

Furthermore, recent studies have shown that mucosal microbiota at different niches exhibit site-specific yet coordinated dysbiosis, suggesting that IgAN may originate from a multi-site mucosal disturbance rather than from gut dysfunction in isolation (84). This notion is consistent with the broader “mucosa–kidney axis” model, in which perturbations in oral or respiratory microbial communities may intensify intestinal inflammation, compromise gut barrier integrity, and ultimately facilitate renal immune injury.

Overall, integrating multiple mucosal compartments into IgAN research may help account for disease heterogeneity, identify triggering factors, and explain variable responses to therapy. Future microbiome studies should incorporate parallel sampling of oral, respiratory, and intestinal microbiota to better delineate their synergistic roles in IgAN pathogenesis.

## 4 The role of mucin-adherent AKK in the gut–kidney axis

**Figure 3:**
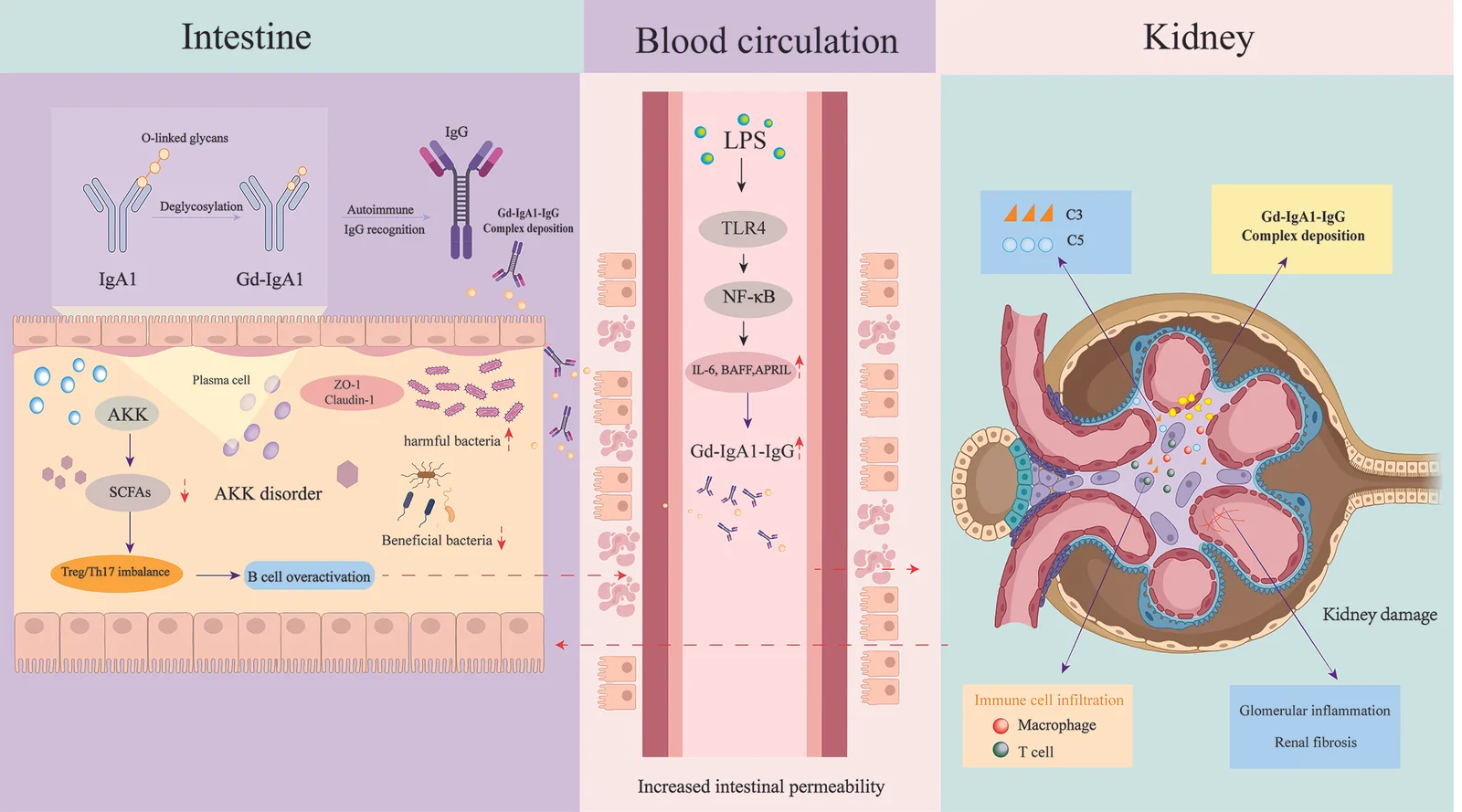
AKK depletion disrupts the gut–kidney axis in IgAN. This schematic outlines a proposed pathogenic cascade whereby depletion of Akk contributes to barrier dysfunction, mucosal immune activation, and subsequent renal immune-complex injury in IgAN. Reduced abundance of Akk—a commensal that degrades mucin and supports epithelial barrier function—induces substantial alterations in intestinal homeostasis. Akk dysregulation leads to thinning of the mucus layer, decreased expression of tight-junction proteins, and increased intestinal permeability, thereby facilitating the translocation of microbial products such as LPS and bacterial DNA. This disruption of the barrier enhances luminal antigen exposure and amplifies local inflammatory signaling. At the same time, reduced production of short-chain fatty acids impairs Treg-mediated immune regulation, while dysbiosis promotes the expansion of pro-inflammatory pathobionts, collectively driving mucosal immune activation. These changes result in enhanced activation of the TLR4–NF-κB signaling pathway, which elevates levels of IL-6, BAFF, and APRIL, and in turn stimulates mucosal IgA1-producing B cells. Based on recent experimental evidence, under these inflammatory conditions, aberrant O-glycosylation of the IgA1 hinge region may occur, potentially contributing to increased Gd-IgA1 production. Gd-IgA1 then crosses the compromised intestinal barrier into the circulation via retrotranscytosis, where it associates with circulating IgG or IgA autoantibodies to form nephritogenic ICs. Due to reduced hepatic clearance, systemic levels of these complexes rise, subsequently affecting the kidneys through the bloodstream. These ICs deposit in the glomerular mesangium, activate complement cascades, and drive mesangial cell proliferation, extracellular matrix expansion, and release of pro-inflammatory cytokines, ultimately resulting in glomerular injury. Renal inflammation further elevates systemic inflammatory tone and promotes the accumulation of uremic toxins, thereby establishing a pathogenic positive feedback loop that sustains gut–kidney axis dysfunction and fuels IgAN progression.

AKK is a Gram-negative anaerobic bacterium that resides in the intestinal mucus layer and utilizes mucin as its primary nutrient source. In healthy individuals, it constitutes approximately 1%–5% of the gut microbial community. This organism contributes to intestinal barrier integrity through the production of SCFAs and by modulating the thickness of the mucus layer (100, 101). However, a growing body of evidence indicates that the role of AKK is highly dependent on the host context. While it exerts protective effects in most metabolic and inflammatory conditions, it may assume a pathogenic function within the specific immunological milieu of IgAN as schematically depicted in **Figure 3** (41, 102).

### 4.1 Biological features and regulation of the intestinal barrier

AKK uses mucin O-glycans as a carbon source and secretes a range of glycan hydrolases, including α-L-fucosidase, β-galactosidase, and sialic acid exohydrolase (103). In addition, its outer membrane protein Amuc_1100 reinforces intestinal epithelial tight junctions via the TLR2 pathway and dampens LPS-induced inflammatory responses (104, 105). In animal models of obesity, type 2 diabetes, and inflammatory bowel disease, administration of AKK has been shown to lower inflammatory markers and improve metabolic parameters (106, 107). In healthy individuals, the abundance of AKK correlates negatively with serum high-sensitivity C-reactive protein levels (108).

### 4.2 The dual role of AKK in IgAN: from protector to pathogen

The hinge region of IgA1 contains multiple O-linked glycosylation sites. Proper galactosylation relies on the coordinated function of core 1 β1,3-galactosyltransferase (*C1GALT1*) and its molecular chaperone *Cosmc* (109). When the *C1GALT1*/*Cosmc* complex is functionally compromised, the terminal glycan chain in the hinge region consists of GalNAc lacking both galactose and sialic acid, resulting in the formation of Gd-IgA1. Genetic studies have confirmed that single-nucleotide polymorphisms in the *C1GALT1* and *Cosmc* genes are associated with susceptibility to IgAN (110). Among them, cytokines such as IL-6 and TGF-β can downregulate C1GALT1 and upregulate ST6GalNAc-II, an aberrant modifying enzyme, through post-transcriptional mechanisms, thereby further exacerbating IgA1 glycosylation abnormalities (109). In IgAN patients, *C1GALT1* expression in B cells is markedly lower than that observed in healthy controls.

Notably, a recent study by Gleeson et al. reported that the relative abundance of mucin-degrading bacteria, including AKK, was significantly higher in the feces of IgAN patients. Using *in-vitro* and *in-vivo* approaches, the authors demonstrated that AKK could directly deglycosylate O-glycans in the human IgA1 hinge region, generating Gd-IgA1; following retrograde endocytosis into intestinal epithelial cells and subsequent entry into the circulation, this deglycosylated IgA1 could deposit in the glomeruli; and in humanized IgA1/CD89 transgenic mice, colonization with AKK worsened the IgAN phenotype. These findings directly connected the enzymatic activity of mucin-degrading bacteria to the four-step pathogenesis model of IgAN, which involves Gd-IgA1 generation, autoantibody recognition, immune complex formation, and glomerular deposition. However, given that this is a single study and the deglycosylation mechanism has not yet been independently replicated, these findings should be regarded as emerging evidence that requires further validation. In contrast, the study by Zhu et al. showed that in IgAN patients, dysbiosis characterized by an increase in *Escherichia-Shigella* and Proteobacteria primarily enhanced BAFF and APRIL expression through the TLR4/MyD88/NF-κB pathway, thereby driving B cells to produce excessive Gd-IgA1 (22). The involvement of AKK in this process, however, remains unclear. Furthermore, in an acute radiation-induced intestinal injury model, supplementation with AKK paradoxically exacerbated mucosal damage, suggesting that its mucin-degrading activity may become detrimental when the intestinal barrier is severely compromised (111).

Based on the evidence outlined above, we propose a context-dependent dual-role hypothesis for AKK in IgAN: (1) in a healthy state or when the intestinal barrier is intact, AKK supports barrier function and suppresses inflammation via the Amuc_1100–TLR2 axis; (2) in the context of IgAN, however, genetic susceptibility (such as *DEFA* or *CARD9* variants) or environmental triggers (infection or dietary factors) may disturb local mucosal immunity. Under these conditions, the mucin-degrading enzyme activity of AKK may excessively expose the O-glycans in the IgA1 hinge region, directly promoting Gd-IgA1 generation. Concurrently, compromised barrier integrity facilitates the entry of this Gd-IgA1 into the circulation.

### 4.3 Potential and challenges of AKK as a therapeutic target

Given the dual nature of AKK, administering live bacteria directly may pose increased risks. At present, alternative approaches under investigation in clinical practice include the following:

1. Pasteurized bacteria: Depommier et al. showed that oral administration of pasteurized AKK was safe in overweight or obese individuals and was associated with signs of metabolic improvement (107). Zhang et al. performed a randomized controlled trial in obese patients with type 2 diabetes and further demonstrated that those with lower baseline abundance of AKK derived more pronounced benefits (112).
2. Outer membrane protein Amuc_1100 or outer membrane vesicles: these probiotic formulations are free of live bacteria and may theoretically circumvent the risk of excessive mucin degradation. In models of sepsis-associated lung injury, pretreatment with Amuc_1100 attenuated tissue damage and remodeled the gut microbiota (113).

For IgAN, future investigations should undertake rigorous clinical trials to ascertain whether inactivated bacteria or probiotic products can restore intestinal barrier function and lower proteinuria without aggravating Gd-IgA1 levels. Given that AKK may directly contribute to Gd-IgA1 production via its deglycosylation activity, any intervention involving this organism must be subjected to comprehensive safety and mechanistic evaluation in dedicated IgAN models (10, 41).

## 5 Clinical translation and treatment strategies

The therapeutic landscape of IgAN is evolving from conventional supportive care, such as RAS blockade, toward more targeted strategies that center on intestinal mucosal immunity, B-cell signaling, and complement pathways within the framework of precision medicine. Sun and Yang proposed “precision targeting of the gut–kidney axis” as a novel therapeutic paradigm for mucosal immunomodulation in IgAN (114). This framework emphasizes a shift from isolated mucosal immune modulation to a multi-target, cross-organ integrated intervention strategy encompassing microbiome regulation, barrier repair, and specific interference with pathogenic IgA1 synthesis or clearance. This conceptual advance provides theoretical guidance for integrating existing and emerging therapeutic approaches. In this section, we highlight interventions that have reached clinical trials or are currently in clinical use, along with gut microbiota-derived biomarkers and prospects for translational application.

### 5.1 Drugs targeting the gut–immune axis with approved or high-level evidence support

#### 5.1.1 Budesonide targeted to the ileal mucosa (Nefecon)

Nefecon is a targeted-release formulation of budesonide that delivers glucocorticoid locally to the terminal ileum, an area rich in Peyer’s patches. Its intended mechanism is to suppress mucosal B-cell activation and Gd-IgA1 production at the source.

In the NEFIGAN trial and the subsequent Phase III NefIgArd study, patients receiving Nefecon for 9 months showed a significant reduction in proteinuria and a slower decline in estimated glomerular filtration rate (eGFR); the renal protective effect was sustained at the 2-year follow-up (71, 115). Biomarker analyses further revealed that Nefecon lowered serum levels of both Gd-IgA1 and anti-Gd-IgA1-IgG antibodies (71). Based on these findings, Nefecon is currently considered the most clearly mechanism-based gut–renal axis-targeted therapy.

#### 5.1.2 Dual endothelin-angiotensin receptor antagonist (Sparsentan)

Sparsentan simultaneously blocks the endothelin A receptor and the type 1 angiotensin II receptor. In the Phase III PROTECT trial, Sparsentan demonstrated a significant reduction in proteinuria compared with irbesartan at 36 weeks, with a favorable safety profile (115).

### 5.2 Targeted therapies directed at B cells and plasma cells (phase II–III clinical trials)

#### 5.2.1 Inhibitors of APRIL and BAFF

Sibeprenlimab, an anti-APRIL monoclonal antibody, significantly reduced proteinuria, stabilized eGFR, and decreased serum Gd-IgA1 levels after 12 months of treatment in a Phase II trial (64).

Atacicept, a TACI-Fc fusion protein that blocks both APRIL and BAFF, demonstrated a significant reduction in proteinuria at 72 weeks along with stable eGFR in the Phase IIb ORIGIN trial (65).

Telitacicept, a TACI-Fc agent approved in China, has shown efficacy in relapsed or refractory IgAN; however, randomized controlled trial evidence continues to accrue (116).

In addition to APRIL/BAFF-targeting biologics, the α4β7–MAdCAM-1 axis has recently emerged as a promising therapeutic target for IgAN. A 2026 multi-omics study published in *Kidney International* provided the most direct evidence to date for the gut–kidney axis in IgAN. By analyzing ileocecal and blood samples from 99 IgAN patients and 121 healthy controls, the study demonstrated that terminal ileum-derived IgA^+^β7^+^ cells—rather than ascending colon-derived cells—are the primary source of Gd-IgA1–producing cells. The gene expression profiles of these cells more closely resembled those of ileum-derived B cells than tonsil-derived B cells. In humanized IgAN mouse models, anti-α4β7 monoclonal antibody treatment reduced β7^+^ cell numbers and Gd-IgA1 levels, induced Peyer’s patch atrophy, and prevented mesangial IgA deposition (117). These findings directly identify the α4β7–MAdCAM-1 axis as a novel therapeutic target and provide precise anatomical guidance for the optimal site of intervention (terminal ileum) in future FMT or probiotic trials.

### 5.3 Exploratory evidence for targeted interventions on gut microbiota

#### 5.3.1 Probiotics and synbiotics

In an IgAN mouse model, Tan et al. administered a probiotic combination comprising *Lactobacillus rhamnosus* and *Lactobacillus bulgaricus* (28). They observed reduced proteinuria, attenuated mesangial injury, and restored intestinal barrier function, along with suppression of the NLRP3 inflammasome in renal tissue and an increased abundance of short-chain fatty acid-producing bacteria in the gut microbiota. However, high-quality randomized controlled trials (RCTs) are currently lacking to demonstrate that probiotics can consistently lower Gd-IgA1 levels in IgAN patients or slow the decline in eGFR (28, 118). Further studies with well-defined bacterial strains and standardized endpoints are warranted.

#### 5.3.2 FMT

Zhi et al. performed an open-label proof-of-concept study in which fecal microbiota capsules were given as an add-on therapy to IgAN patients already receiving ACEI/ARB treatment (27). The results indicated that FMT was generally well-tolerated, with 24h urinary protein levels showing a significant reduction from baseline at the 1- to 3-month follow-up. Peripheral B-cell counts also declined, and these changes correlated with shifts in gut microbial composition and metabolite profiles. Case reports have additionally suggested that FMT may lower proteinuria in patients with refractory IgAN (119). However, these studies have limitations due to their small sample sizes and the lack of a control design. They are either open-label or non-controlled. At present, FMT remains at the proof-of-concept stage, and rigorous RCTs are needed to confirm its long-term efficacy and safety (27, 29).

#### 5.3.3 Traditional Chinese medicines: exploratory evidence based on intestinal mechanisms

Several traditional Chinese medicine formulations have been shown in animal models to attenuate renal injury in IgAN through protection of the intestinal barrier, modulation of the gut microbiota, and inhibition of the TLR4/NF-κB pathway. For example, Shenqi Yishen Granules reduced proteinuria and mesangial IgA/C3 deposition in *ITGAM* -IRES-hCD89 transgenic mice, with the underlying mechanism involving suppression of the TLR4/MyD88/NF-κB and IL-6/JAK2/STAT3 pathways (120). Similarly, Zhenwu Decoction ameliorated gut microbial dysbiosis and colonic tissue injury in IgAN rats (121).

However, most of these investigations remain at the preclinical stage and lack validation from standardized multicenter RCTs. Future efforts should prioritize the identification of active constituents, standardization of formulations, and elucidation of the causal relationship with the gut microbiota.

### 5.4 Gut microbiota as biomarkers: diagnostic and predictive applications

#### 5.4.1 Biomarkers for diagnosis

A random forest model using fecal 16S rRNA data can discriminate IgAN from healthy controls, achieving an area under the curve (AUC) of 0.96 to 0.99 (122). A multi-omics model that integrates metagenomic and metabolomic profiles can differentiate IgAN from healthy controls (AUC 0.919) and from membranous nephropathy (AUC 0.912) (89). These models remain at the research stage and have not yet been translated into commercially available clinical assays.

#### 5.4.2 Biomarkers for prognosis and treatment response prediction

In a prospective cohort study of 55 patients, Dong et al. found that baseline abundances of *Pseudomonas* and *Escherichia-Shigella* were significantly higher in patients who failed to respond to 6 months of immunosuppressive therapy, and a prediction model based on these features yielded an AUC of 0.910 (123). This suggests that gut microbiota profiling may guide stratified treatment approaches. In addition, baseline gut microbial composition may correlate with responses to Nefecon or other targeted therapies, although this requires prospective validation (124).

### 5.5 Microbiome-based precision medicine: challenges and future directions

Although the lines of evidence reviewed above highlight the considerable promise of the gut microbiota in IgAN, translating these findings into precision medicine continues to face substantial hurdles. First, heterogeneity remains a major issue. Microbial profiles differ considerably across patients from diverse geographic, age, and dietary backgrounds, which complicates the identification of universally applicable biomarkers (125). Second, causality remains uncertain. The majority of current studies are cross-sectional or rely on short-term follow-up, which limits the ability to differentiate between causal effects and mere associations (126). Third, intervention specificity is inadequate. Probiotic formulations and FMT protocols have yet to be standardized with respect to bacterial strains, dosage, and treatment duration (118). Fourth, long-term safety data are lacking. FMT carries potential risks, including the transmission of antibiotic-resistant organisms and the induction of metabolic disturbances (127).

In the future, priority should be given to multicenter, prospective, RCTs that adopt standardized endpoints, including proteinuria remission, eGFR slope, and Gd-IgA1 kinetics, while collecting multi-omics data from fecal, blood, and urine samples. Such efforts will facilitate the development of predictive models linking microbial features to treatment response and support the design of microbiota-based adjunctive therapies, such as specific prebiotics or engineered bacterial strains (27, 41, 64).

## 6 Challenges, research gaps, and future directions

### 6.1 Methodological challenges and limitations

#### 6.1.1 The gut microbiota is highly heterogeneous and dynamic

Its composition is shaped by multiple factors, including diet, age, geographic location, and medication use. A single time-point stool sample is therefore inadequate to capture the long-term, stable, disease-relevant features of the microbial community (125, 128).

#### 6.1.2 Study design shortcomings are prevalent

Most available investigations are cross-sectional in nature, involve small sample sizes, and are performed at single centers. They also lack extended follow-up (≥1–2 years) and standardized protocols for sample collection, sequencing, and data analysis (42, 126).

#### 6.1.3 Technical limitations

The resolution of 16S rRNA sequencing is limited, making it difficult to reach the strain level and functional prediction; the costs of metagenomics and metabolomics are high, and they are still not widely available (129, 130).

#### 6.1.4 Insufficient control for confounding factors

Concurrent use of medications (such as RAS blockers, immunosuppressants, and antibiotics) and comorbidities (hypertension and diabetes) may independently affect the microbiota, leading to false associations (126, 131).

### 6.2 Key knowledge gaps

Despite the substantial progress reviewed above, several key knowledge gaps remain unresolved.

#### 6.2.1 Certainty of causal relationship

Although MR studies and FMT experiments in animals support dysbiosis as a driving factor, the long-term and dynamic causal sequence in humans has yet to be established. Longitudinal time-series investigations are required to monitor microbial shifts during the early phases of disease, including the preclinical period, and to align these changes chronologically with IgAN onset (25, 132).

#### 6.2.2 Unclear molecular mechanisms of IgA1 O-glycosylation

The regulation of IgA1 O-glycosylation by the microbiota is poorly understood. In addition to the deglycosylase activity of AKK reported in a recent study, other bacterial enzymes may also be involved. It also remains unknown whether host glycosyltransferases such as *C1GALT1* and ST6GalNAc-II are modulated by microbial metabolites (109, 110). These questions warrant further mechanistic exploration.

#### 6.2.3 Unidentified upstream microbial ligands

The identity of the upstream microbial ligands that engage the TLR4/TLR9–BAFF/APRIL pathway is not yet clear. Potential candidates include LPS, flagellin, or other PAMPs, but this remains to be determined (22, 60).

#### 6.2.4 Unclear contribution of AhR signaling

The direct contribution of AhR signaling to renal injury in IgAN is still unclear. In particular, it is uncertain whether IS-induced trained immunity via AhR occurs in monocytes or macrophages of IgAN patients (54).

#### 6.2.5 Limited understanding of complement–microbiota interplay

The interplay between complement activation and the gut microbiota remains poorly defined. It is not known whether intestinal-derived PAMPs or metabolites directly modulate the alternative and lectin complement pathways (66, 67).

#### 6.2.6 Translational gaps between animal models and human disease

Although humanized mouse models such as α1KI-CD89Tg partially recapitulate IgAN, the complexity of the human microbiota, interspecies immune differences, and the prolonged disease course in patients preclude complete replication. Consequently, many interventions that show efficacy in animal models, including probiotics and FMT, exhibit considerably weaker effects in humans than anticipated (24, 28).

#### 6.2.7 Synergistic effect of mucosal microbiota at multiple sites

The microbiota in the oral, pharyngeal, and intestinal regions may interact with each other and jointly drive the systemic mucosal immune response. Currently, there is a lack of studies that simultaneously collect data from multiple sites and conduct longitudinal tracking (84, 133).

#### 6.2.8 Lack of predictive biomarkers

Despite the promising performance of diagnostic models, microbiota-based biomarkers for predicting disease progression, response to immunosuppressive therapy, or recurrence risk remain scarce and require further development (119, 134).

Addressing these knowledge gaps is critical for translating mechanistic insights into clinical practice. Notably, recent 2026 multi-omics advances have identified the terminal ileum as the primary source of Gd-IgA1-producing cells, yet the safety and efficacy of targeting the α4β7–MAdCAM-1 axis in human IgAN remain to be established (117). Similarly, the precise mechanisms by which the gut virome contributes to IgAN pathogenesis, the exact signaling pathways through which microbial metabolites such as sphingosine-1-phosphate and acetate mediate renal injury, and the translational potential of engineered *AKK* strains all require further functional validation in IgAN-specific models and clinical cohorts (83, 95, 96, 135).

### 6.3 Future research directions and priorities

#### 6.3.1 Mechanism research

To address these knowledge gaps, several lines of mechanistic investigation should be prioritized. First, single-cell sequencing and spatial transcriptomics should be employed to map the active cellular components and microenvironments associated with microbiota-related signaling pathways—including TLR4, BAFF/APRIL, and AhR—within intestinal and renal tissues. Second, IgA-seq or bacterial–IgA co-analysis should be used to directly identify gut bacterial species that bind Gd-IgA1 and to characterize their functional gene repertoires (44). Third, germ-free or limited-bacteria humanized mice should be constructed, colonized with specific strains or combinations of microbiota, to verify their contributions to IgA glycosylation, immune complex formation, and kidney injury in a stepwise manner. Fourth, the complement–microbiota axis should be investigated by administering complement inhibitors, such as iptacopan, in animal models to clarify the causal link between microbial dysbiosis and complement activation (68).

#### 6.3.2 Clinical translation and trial design

To translate mechanistic insights into clinical practice, well-designed clinical studies are urgently needed.

1. Large-scale prospective cohort studies: Enroll high-risk populations, including individuals with a family history of IgAN, a history of IgA vasculitis, or recurrent tonsillitis. Collect fecal, oral swab, and blood samples at 3- to 6-month intervals over a 2- to 5-year follow-up period to monitor the onset and progression of IgAN and to establish the temporal order and causal contribution of microbiome alterations.
2. Standardization of RCTs: For probiotic, FMT, or synbiotic interventions, a placebo-controlled, double-blind, multicenter design should be employed, with standardized bacterial strains, dosages, and treatment courses. Primary endpoints should encompass proteinuria remission, defined as a ≥50% reduction in the urine protein-to-creatinine ratio, changes in the eGFR slope, reduction in Gd-IgA1 levels, and a composite renal outcome that includes a ≥30% decline in eGFR, ESRD, and death. Safety endpoints should cover serious adverse events, infections, malignancy risk, and the potential transmission of antimicrobial resistance genes from the microbiome.
3. Microbiome-guided stratified treatment: In clinical trials of agents such as Nefecon, Sibeprenlimab, and Atacicept, baseline microbiome samples should be collected prospectively. The microbial profiles of responders and non-responders should be compared, and a predictive model developed and subsequently validated in independent patient cohorts (123, 124).
4. Integration of multi-omics and artificial intelligence: By combining fecal metagenomics, metabolomics, host genomic data (including GWAS-derived risk scores), proteomics, and clinical information, a classification system for IgAN subtypes should be developed to inform personalized treatment strategies (132).

#### 6.3.3 Emerging therapeutic strategies

Beyond the interventions already in clinical development, several emerging therapeutic strategies warrant further investigation. First, engineered bacteria and synthetic microbial consortia could be designed to achieve stable gut colonization and sustained production of short-chain fatty acids or antimicrobial peptides, while minimizing the risk of excessive mucin degradation. Second, probiotic preparations—such as pasteurized inactivated AKK or Amuc_1100 proteins—require safety and efficacy verification specifically in IgAN (107, 112). Third, adsorbents and enzyme inhibitors represent another promising avenue: oral activated carbon (AST-120) can adsorb toxin precursors such as indole, although findings from large-scale clinical trials have been inconsistent (136); selective inhibitors of intestinal tryptophanase can lower IS levels, but they have not yet been evaluated in IgAN trials (36). Fourth, antigen-specific degradation: agents such as BHV-1400 can selectively degrade Gd-IgA1 without causing systemic immunosuppression and are currently in early-stage development (137).

## 7 Conclusion

The pathogenesis of IgAN is complex and involves the interaction of multiple factors, among which gut microbiota dysbiosis plays a critical role. Accumulating evidence indicates that microbial alterations promote Gd-IgA1 production and exacerbate renal injury through disruption of the intestinal barrier and activation of systemic immune responses. The gut–kidney axis concept provides a novel framework for understanding IgAN pathophysiology, highlighting the intimate link between intestinal homeostasis and renal function.

Despite substantial progress, several key questions remain unresolved, including the causal role of gut dysbiosis in aberrant IgA production, the precise molecular mechanisms involved, and the optimal strategies for translating microbiota-targeted interventions into clinical practice. Future research should prioritize these issues through well-designed mechanistic studies and clinical trials. Individualized strategies—tailored to specific gut microbiota profiles—may offer novel therapeutic avenues for IgAN patients and ultimately improve clinical outcomes.
